# Impact of remote ischemic preconditioning preceding coronary artery bypass grafting on inducing neuroprotection (RIPCAGE): study protocol for a randomized controlled trial

**DOI:** 10.1186/1745-6215-15-414

**Published:** 2014-10-27

**Authors:** Hrvoje Gasparovic, Tomislav Kopjar, Milan Rados, Alan Anticevic, Marko Rados, Branko Malojcic, Visnja Ivancan, Tea Fabijanic, Maja Cikes, Davor Milicic, Vladimir Gasparovic, Bojan Biocina

**Affiliations:** Department of Cardiac Surgery, University Hospital Center Zagreb, University of Zagreb, Kispaticeva 12, 10 000 Zagreb, Croatia; Croatian Institute for Brain Research, School of Medicine University of Zagreb, Zagreb, Croatia; Departments of Psychiatry and Psychology, Yale University School of Medicine, New Haven, USA; Department of Radiology, University Hospital Center Zagreb, University of Zagreb, Zagreb, Croatia; Department of Neurology, University Hospital Center Zagreb, University of Zagreb, Zagreb, Croatia; Departments of Cardiology and Internal Medicine, University Hospital Center Zagreb, University of Zagreb, Zagreb, Croatia

**Keywords:** coronary artery bypass grafting, functional, magnetic resonance imaging, neurocognitive outcome, remote ischemic preconditioning

## Abstract

**Background:**

Neurological complications after cardiac surgery have a profound impact on postoperative survival and quality of life. The increasing importance of strategies designed to improve neurological outcomes mirrors the growing risk burden of the contemporary cardiac surgical population. Remote ischemic preconditioning (RIPC) reduces adverse sequelae of ischemia in vulnerable organs by subjecting tissues with high ischemic tolerance to brief periods of hypoperfusion. This trial will evaluate the neuroprotective effect of RIPC in the cardiac surgical arena, by employing magnetic resonance imaging (MRI) and neurocognitive testing.

**Methods:**

Patients scheduled for elective coronary artery bypass grafting with the use of cardiopulmonary bypass will be screened for the study. Eligible patients will be randomized to undergo either a validated RIPC protocol or a sham procedure. The RIPC will be induced by inflation of a blood pressure cuff to 200 mmHg for 5 minutes, followed by a 5-minute reperfusion period. Three sequences of interchanging cuff inflations and deflations will be employed. Neurocognitive testing and MRI imaging will be performed preoperatively and on postoperative day 7. Paired pre- and postoperative neurocognitive and neuroimaging data will then be compared. The primary composite outcome measure will consist of new ischemic lesions on brain MRI, postprocedural impairment in brain connectivity on resting-state functional MRI (rs-fMRI), and significant new declines in neurocognitive performance. The secondary endpoint measures will be the individual components of the primary endpoint measures, expressed as continuous variables, troponin T release on postoperative day 1 and the incidence of major adverse cardiovascular events at 3 months postoperatively. Major adverse cardiovascular events, including accumulating cardiovascular mortality, stroke, nonfatal myocardial infarction, and rehospitalization for ischemia, will form a composite endpoint measure.

**Discussion:**

This trial will aim to assess whether RIPC in patients subjected to surgical myocardial revascularization employing cardiopulmonary bypass initiates a neuroprotective response. Should the results of this trial indicate that RIPC is effective in reducing the incidence of adverse neurological events in patients undergoing coronary artery bypass grafting, it could impact on the current standard of care.

**Trial registration:**

ClinicalTrials.gov NCT02177981.

## Background

Neurological impairment following coronary artery bypass grafting may take on the form of stroke or postoperative cognitive dysfunction. The former is rare, but potentially devastating. However, a decline in attention, memory, or fine motor skills can frequently be documented by sensitive neurocognitive testing [1]. Postoperative deterioration in neurocognitive capacity is often discrete, but exerts an adverse impact on the quality of life. Its reversibility is subject to interpatient variability, with 25% of patients still exhibiting signs of cognitive impairment 6 months after surgery [1, 2].

Ischemic preconditioning exploits the endogenous protective potential against sustained ischemia incurred by brief episodes of ischemia and reperfusion [3]. The phenomenon is probably triggered by both neuronal and humoral pathways [3]. In an experimental setting, transfer of coronary effluent from an ischemically preconditioned rabbit to an ischemic-preconditioning-naïve animal reduced myocardial infarct size in the latter [4]. This observation consolidated the hypothesis of a humoral protective mediator [4]. This hypothesis was corroborated by the induction of protection in a denervated donor heart by limb ischemia [5]. The fact that ischemic-preconditioning-induced organ protection was inhibited by a ganglion blocker, however, gave credence to the idea of the existence of underlying neural pathways [3].

Remote ischemic preconditioning (RIPC) denotes the concept by which brief episodes of sublethal ischemia in tissues with high ischemic tolerance stimulate protection in distant organs from a subsequent severe insult [6]. It is conceptually much simpler to induce than conventional ischemic preconditioning, thereby expanding its clinical applicability. Several reports have demonstrated that RIPC protocols lead to reductions in myocardial injury following cardiac surgery [7, 8]. Thielmann *et al.* [9] recently showed that RIPC induced by upper limb ischemia in patients undergoing elective coronary artery bypass grafting led to a reduction in troponin T release, which mirrored an observed reduction in mortality (ratio, 0.27; 95% confidence interval, 0.08 to 0.98; *P* = 0.046).

The relative paucity of effective management options to treat neurological dysfunctions once they have become clinically manifest provides a strong impetus for defining strategies that reduce their incidence [10]. Ischemic preconditioning has been shown to induce ischemic tolerance in the brain via key survival signaling pathways, which promote synaptic and mitochondrial adaptations [11]. These modifications reduce the adverse impact of excitotoxicity, which is pivotal to ischemic neuronal injury [11]. Ischemic preconditioning produces two windows of protection. The early window of protection follows rapidly after the event that induced it [10], while delayed preconditioning occurs after a 24-hour delay [10]. The latter is protein synthesis-dependent, with subsequent protection extending for up to 96 hours. The benefits of early preconditioning are that it can be relied upon to promote organ protection immediately prior to an index event carrying a risk of brain injury [10]. In an experimental model, RIPC has been found to exert a beneficial effect on multiple variables acting as surrogate markers of brain injury following prolonged circulatory arrest [12]. These included a reduction in brain lactate release, a faster recovery of electroencephalographic activity and superior quantitative behavioral scores [12].

Magnetic resonance imaging (MRI) allows for sensitive delineation of the anatomical substrate underlying ischemic injury [13]. Its potential to detect clinically silent brain injury has been clearly recognized [14]. Specifically, hyperintensity on diffusion-weighted imaging, together with a decrease in the apparent diffusion coefficient, is effective in demarcating the core of acute ischemic lesions [13]. Combinations of different MRI signals provide complementary information on the evolution of organic neuropathology [13]. Resting-state functional MRI (rs-fMRI) is a novel instrument that focuses on functional brain connectivity, rather than attempting to define structural pathology [15]; it harbors the potential to identify the presence of anatomically divergent regions that operate in synchrony as large-scale neural networks [16]. These anatomically distributed regions act in concert to determine a variety of complex behavioral patterns.

In our study, coronary surgery using cardiopulmonary bypass will be considered the index deleterious event. The mechanisms of neurologic injury following cardiopulmonary bypass include ischemia, inflammation, cerebral edema, and hyperthermia [1]. Brain ischemia occurring during cardiac surgery might be the consequence of embolic showers consistently shown on transcranial Doppler during episodes of aortic manipulation, or procedure-associated hypoperfusion [1, 14].

Strategies designed to protect end-organ function are paramount in current cardiac surgical practice. The convergence of the ever-increasing patient-risk profile with the growing complexity of modern cardiac surgical procedures underscores the importance of defining algorithms that will maintain acceptable clinical outcomes.

We hypothesized that the neuroprotective potential of RIPC could translate into an improvement in neurological imaging or functional outcomes among cardiac surgical patients.

## Methods

### Study population

**Figure 1:**
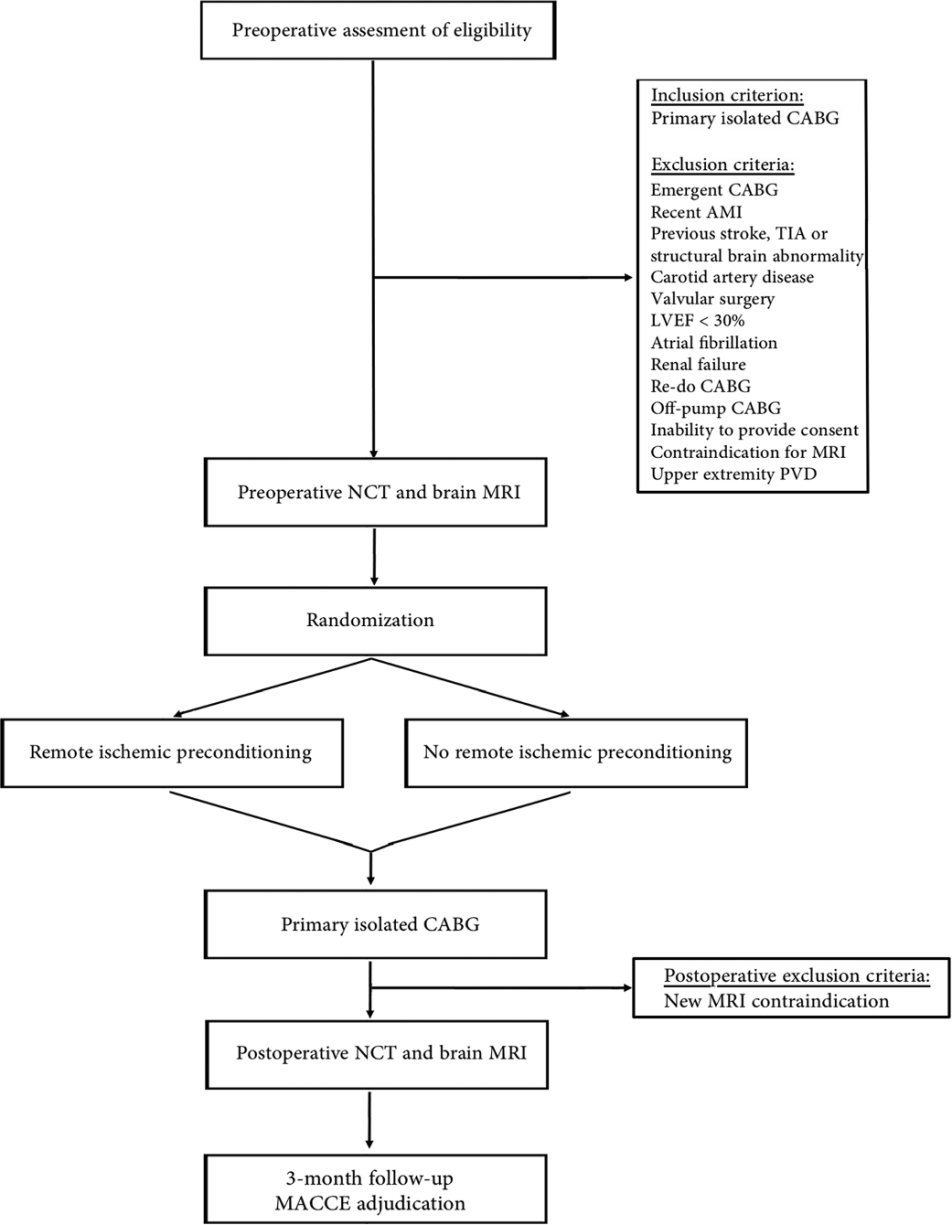
**Flowchart depicting the screening, recruitment and randomization algorithm.** AMI, acute myocardial infarction; CABG, coronary artery bypass grafting; LVEF, left ventricular ejection fraction; MACCE, major adverse cardiac and cerebrovascular event; MRI, magnetic resonance imaging; NCT, neurocognitive testing, PVD, peripheral vascular disease; TIA, transitory ischemic attack.

The study is designed as a single-center, prospective, randomized, double-blind controlled trial (Figure 1). Adult patients with multivessel coronary disease undergoing primary, elective coronary artery bypass grafting at the University Hospital Center Zagreb will be eligible for study enrollment. Following the process of primary triage, they will be approached by a study nurse who will provide detailed information about the trial. Written informed consent will be obtained from all patients prior to enrollment. Preoperative and postoperative exclusion criteria are summarized in Table 1.

**Table 1:** **Study inclusion and exclusion criteria**

*Inclusion criteria*	*Rationale for inclusion*
Adult patients (18 to 80 years) undergoing primary coronary artery bypass grafting with the use of cardiopulmonary bypass	Population of interest
***Preoperative exclusion criteria***	***Rationale for exclusion***
Emergent coronary artery bypass grafting	Higher-risk profile
Recent acute myocardial infarction	Concurrent indication for dual antiplatelet therapy
History of stroke or transitory ischemic attack	Prior neurological condition
Structural brain abnormalities	Prior neurological condition
Carotid artery disease	Ineligible for study enrollment
Valve surgery	Ineligible for study enrollment
Left ventricular ejection fraction <30%	Higher-risk profile
Atrial fibrillation	Higher-risk of neurological complications
Renal failure	Higher-risk profile
Repeat coronary artery bypass grafting	Higher-risk profile
Off-pump coronary artery bypass grafting	Avoidance of cardiopulmonary bypass
Inability to provide consent	Ineligible for study enrollment
Contraindications for MRI	Ineligible for study enrollment
Peripheral vascular disease of upper extremities	Contraindication for remote ischemic preconditioning
***Postoperative exclusion criteria***	***Rationale for exclusion***
New-onset contraindication for MRI	Protocol violation
Hemodynamic instability	Inability to complete follow-up MRI

MRI, magnetic resonance imaging.

### Ethics

The ethics committee of the University Hospital Center Zagreb evaluated and approved the conduct of this study (Registration No. 380-59-10106-13-195/292) in December 2013. Ethical standards in line with the Declaration of Helsinki involving research on human subjects will be strictly adhered to. This protocol complies with CONSORT guidelines [17].

### Sample size

The estimation of the approximate sample size for this study was based on an exact sign (binomial) test, assuming an effect size of 0.20. To account for potential postoperative contraindications for MRI and study consent withdrawals, an estimated 70 patients will be required to test the null hypothesis with an α value of 0.05 and a power of 0.90 [18].

### Allocation procedure

Randomization will be based on computer generated coding. Codes will be kept in sealed envelopes. These will be opened in the operating room by an anesthesiologist not involved in subsequent data accumulation or analysis. Patients randomized to the intervention arm will be subjected to brief periods of upper limb ischemia designed to induce RIPC, as described later. Patients assigned to the control arm will receive no ischemic preconditioning. Surgeons and intensive care physicians will be unaware of treatment allocation.

### Intervention and control protocols

Remote ischemic preconditioning will be induced by alternating periods of upper limb ischemia and reperfusion. Transient arm ischemia will be produced by inflating a blood pressure cuff to 200 mmHg for 5 min, and then deflating it for 5 min. This sequence will be repeated three times after the induction of anesthesia and prior to the surgical incision. Our intervention protocol has been used previously and constitutes a validated protocol with beneficial effects exerted at the myocardial level [9]. Patients in the control group will also have a blood pressure cuff placed, but it will not undergo the aforementioned inflation-and-deflation cycles.

### Myocardial revascularization

Morphine and diazepam will be administered to patients preoperatively. Induction and maintenance of anesthesia will rely on a combination of midazolam, sufentanil, pancuronium bromide, and sevoflurane. Propofol will be avoided, owing to its potential for inhibiting RIPC-inducing pathways [9].

Coronary revascularization will be performed via a median sternotomy with the use of cardiopulmonary bypass. Systemic heparinization with a target activated clotting time >450 s will be utilized. Both distal coronary and proximal anastomoses will be performed during a single period of aortic cross-clamping, thereby reducing the embolic burden related to aortic manipulation. Myocardial protection will be based on administration of both antegrade and retrograde cardioplegia. In the immediate period after cardiopulmonary bypass, a goal-oriented strategy targeting a cardiac index of 2.2 l/(min m^2^) will be employed. Interventions to attain this goal will include optimization of preload and, if necessary, inotropic support. The preferred inotropic agent in our institution is dobutamine. In the unlikely event that further escalation of vasoactive support is required, our armamentarium also includes phosphodiesterase 3 inhibitors, intra-aortic balloon pumps, and mechanical circulatory assistance. Full heparin reversal with protamine will be employed.

### Blood sampling

Venous blood samples for the measurement of troponin T will be taken on postoperative day 1. We will employ the Elecsys® Troponin T high-sensitive assay (Roche, Basel, Switzerland).

### Perioperative management

Postoperative medications will typically include a beta-blocker, a hydroxy-methyl-glutaryl-CoA reductase inhibitor and a diuretic. Aspirin (300 mg) will be administered within 6 hours of surgery, provided that the chest tube output is minimal. The use of angiotensin-converting-enzyme inhibitors postoperatively will be individualized and tailored to effect. Peptic ulcer prophylaxis will be universally implemented in the perioperative period, and continued for six weeks.

### Neurocognitive testing

Neurocognition in study patients will be evaluated using the Montreal Cognitive Assessment and the Trail Making Test. Neurocognitive testing will be performed preoperatively and on postoperative day 7. The Montreal Cognitive Assessment is a screening instrument for mild cognitive dysfunction, but it has also been used to assess cognitive functions in various neurological disorders, such as stroke, Parkinson’s disease, and multiple sclerosis [19–21]. It is suitable for the assessment of both distributed and focal cognitive domains, such as attention and concentration, executive functions, memory, language, visuoconstructional skills, conceptual thinking, calculation, and orientation. While the maximum test result is 30 points, a score of 26 is at the lower extreme of normal. As the patients in this study will be screened twice in a short period of time, we will use two versions of the test to negate the possible influence of a learning curve. A validated Croatian-language version is available for clinical and research purposes [22]. The Trail Making Test is a commonly employed neuropsychological instrument for evaluating visual attention, speed of processing and executive functioning [23]. It consists of two separate tasks, which evaluate the subject’s ability to alternate between cognitive categories [24]. In Part A, the subject is required to link randomly assigned numbers in ascending order. The goal in Part B is to link alternate numbers and letters in a pre-determined order (that is, 1-A-2-B…). The personnel responsible for neurocognitive testing will be unaware of the individual patient’s allocation to either the intervention or control arms.

### Neuroimaging

A comprehensive set of MRI sequences will be used for the analysis and quantification of structural and functional brain impairment following a standardized cardiac surgical procedure (Table 2). Patients with significant structural abnormalities identified on preoperative brain MRI (such as hydrocephalus, neurodevelopmental disorders, or brain tumors) will be excluded from the study. Magnetic resonance imaging will be performed twice in all patients (before cardiac surgery and on postoperative day 7) on a 3 T MR scanner (Magnetom TrioTim; Siemens, Munich, Germany) using a 32-channel head coil. To detect new structural ischemic lesions, pre and postoperative MRI exams will be compared for each patient. Fluid attenuated inversion recovery sequences (TR, 9000 ms; TE, 87 ms; voxel size, 0.9 × 0.9 × 4 mm,; field of view, 230 × 183; matrix, 204 × 256; flip angle, 130°) will be used to detect ischemic lesions and diffusion-weighted imaging sequences (TR, 3100 ms; TE, 92 ms; voxel size, 1.8 × 1.8 × 5 mm; field of view, 230 × 230; matrix, 128 × 128; b1,= 0 s/mm^2^; b2, 500 s/mm^2^; b3, 1000 s/mm^2^), together with calculated apparent diffusion coefficient maps, will be used to differentiate between old and new ischemic lesions. Regions of diffusion-weighted imaging hyperintensity and decreases in apparent diffusion coefficient correlate with acute ischemic brain injury [13], and will constitute a component of the primary endpoint measure. The volumes of new ischemic lesions will be calculated using Analyze 8.1 software (Mayo Clinic, Rochester, MN, USA). For rs-fMRI, gradient-echo echoplanar imaging sequences sensitive for blood-oxygen level dependent contrast will be applied (TR, 3000 ms; TE, 31 ms; voxel size, 3.4 × 3.4.3 mm; field of view, 220 × 220; matrix, 64 × 64; flip angle, 90°) for a period of 10 minutes and 8 seconds. Functional image data will be co-registered with high-resolution magnetization-prepared rapid acquisition gradient-echo T1 sequences (TR, 1900 ms; TE, 2.52 ms; voxel size, 1 × 1 × 1 mm; field of view, 250 × 250; matrix, 246 × 256, flip angle, 9°). The advantage of rs-fMRI lies in its ability to detect increases in the blood-oxygen level dependent contrast, as a measure of neural activity. Blood-oxygen level dependent signal variability is a surrogate measure of integrated synaptic activity [16]. All preprocessing, quality assurance, structural, and functional connectivity analyses will follow prior validated and published approaches that have been applied to clinical populations [25, 26]. Briefly, we will perform the following preprocessing steps for all blood-oxygen level dependent images, as previously described [25, 26]: (i) slice-time correction, (ii) first five images removed from each run, (iii) rigid-body motion correction, (iv) 12-parameter affine transform of the structural image to the Talairach coordinate system, and (v) coregistration of volumes to the structural image with 3 × 3 × 3 mm resampling.

**Table 2:** **Summary of the MRI neuroimaging portfolio**

***MRI imaging sequences***	***Diagnostic target***
T1, T2, proton density signal weighting	Ischemic or hemorrhagic brain lesions.
	Structural brain abnormalities (hydrocephalus, neurodevelopmental disorders, brain tumors).
Magnetization-prepared rapid acquisition gradient-echo (three-dimensional)	Structural brain abnormalities.
	Anatomical three-dimensional template for subsequent rs-fMRI coregistration.
Fluid attenuated inversion recovery	Ischemic lesion of the brain parenchyma (acute, subacute, and chronic).
Diffusion-weighted imaging	Discrimination between acute, subacute, and chronic ischemic lesions, based on reduced diffusibility in acute or subacute lesions presenting as high signal on diffusion-weighted imaging sequence and low signal on calculated apparent diffusion coefficient maps.
rs-fMRI	Functional brain connectivity within neural networks. Comparison of functional connectivity patterns before and after operation.

MRI, magnetic resonance imaging; rs-fMRI, resting-state functional magnetic resonance imaging.

In addition, all blood-oxygen level dependent images will pass stringent quality assurance criteria to ensure that all data were of high and comparable quality across groups: (i) signal-to-noise ratios >100, computed by obtaining the mean signal and standard deviation for a given slice across the blood-oxygen level dependent run, while excluding all nonbrain voxels across all frames [1]; (ii) no blood-oxygen level dependent run with a single frame movement greater than one functional voxel; (iii) movement scrubbing [27, 28]. Lastly, to remove potential sources of spurious signal in resting-state data, additional preprocessing steps will be completed, according to standard practice [29]: all blood-oxygen level dependent time-series underwent high-pass (0.009 Hz) and low-pass (0.08 Hz) temporal filtering, nuisance signal removal from ventricles and deep white matter, global signal regression, six rigid-body motion correction parameters, and first derivatives using in-house MatLab tools [30].

### Outcome definitions

The primary composite outcome will consist of new ischemic lesions on brain MRI, impairment in brain connectivity on resting-state functional MRI (rs-fMRI) and postoperative cognitive dysfunction. Postoperative cognitive dysfunction will be defined as a decrease of at least one standard deviation in at least two components of neurocognitive testing.

The secondary endpoint measures will be:

1. Volumetric quantification of areas of new ischemic lesions on structural brain MRI.
2. Changes between pre- and post-rs-fMRI, expressed as continuous variables and compared among the intervention and control arms.
3. Percentage declines in individual neurocognitive tests.
4. Troponin T release on postoperative day 1.
5. Incidence of major adverse cardiovascular events at 3 months postoperatively.

Major adverse cardiovascular events will comprise a composite endpoint measure, consisting of cardiovascular mortality, stroke, nonfatal myocardial infarction, and rehospitalization for ischemia. Perioperative myocardial infarction will be adjudicated in accordance to the definition supplied by the Joint European Society of Cardiology/American College of Cardiology Foundation/American Heart Association/World Heart Federation Task Force for the Redefinition of Myocardial Infarction [31]. Briefly, a troponin T value ten times the 99th percentile of the upper reference limit, coupled with electrocardiogram changes, new-onset coronary artery (or graft) occlusion, or imaging evidence of new wall motion abnormalities will meet the criteria for perioperative myocardial infarction [31]. Stroke will be a defined on the basis of either a new focal deficit lasting >24 hours or an imaging study suggestive of a new lesion in patients with prompt neurological recovery [32].

### Statistical analysis

The frequency distributions of primary and secondary study endpoints will be compared across the groups using 2 × 2 contingency tables. Continuous data will be presented as mean values ± standard deviation or medians with their respective interquartile range. Categorical variables will be presented as absolute numbers with percentages. Measures of association of categorical data will be derived from Fisher’s exact test. Analysis of continuous data will rely on the Mann-Whitney *U* test or Student’s *t* test. Comparisons of functional connectivity between regions of interest on pre- and postoperative rs-fMRI will be performed with the Wilcoxon matched-pairs test. Correlations between multiple variables of interest will be examined using Spearman’s or Pearson’s correlation coefficients. The choice between parametric and nonparametric statistical tools will be based on the normality of distribution. A two-tailed *P* value of less than 0.05 will be considered significant for all deployed statistical calculations. The data will be processed using the IBM SPSS Statistics software package (version 20.0; Somers, NY, USA).

## Discussion

Alternating cycles of nonlethal ischemia and reperfusion induce an endogenous response that renders tissues more tolerant to a subsequent prolonged ischemic ictus [3]. In contrast with ischemic preconditioning, which is essentially restricted to the experimental arena, RIPC has the capacity for translation into the clinical domain [9]; it induces ischemic tolerance in distant organs by provoking ischemia in tissues that are both easily accessible and resistant to noxious stimuli. In this prospective randomized trial, RIPC will be evaluated as a neuroprotective strategy in the cardiac surgical arena. We believe that the high frequency of discrete neurological dysfunction following cardiac surgery, coupled with the proven benefit of RIPC on the induction of ischemic tolerance in other organs, constitutes a sound basis for this research. A strategy that would increase ischemic tolerance within the ischemic penumbra could potentially yield superior neurological outcomes.

In this prospective randomized study, a homogenous cardiac surgical patient population will be subjected to a validated RIPC protocol. Previous research has focused on the reduction of myocardial ischemic injury after cardiac surgery induced by RIPC [1]. To the best of our knowledge, this is the first trial that will specifically address the neuroprotective value of this strategy in patients undergoing surgical myocardial revascularization. The comprehensive battery of brain MRI tools that will be used, coupled with neurocognitive testing, will allow for the identification of discrete neurologic injuries, thereby increasing the trial’s diagnostic sensitivity.

The etiology of brain injury following cardiac surgery is multifaceted, and not all of its potential triggers can be addressed in a single trial. Our study will focus on a strategy hypothesized to reduce the impact of ischemia on end-organ performance.

While the trial will enroll patients from the lower-risk stratum of the contemporary cardiac surgical practice, the fact that the pathophysiology of brain ischemia is no different in higher-risk patients will allow for the results of this trial to be generalized onto a wider surgical cohort. Should the preoperative implementation of a RIPC protocol result in improvement in postoperative neurologic outcomes, the RIPCAGE trial could meaningfully impact on the standard of care in cardiac surgical patients.

## Trial status

Patient recruitment for the study is currently ongoing.

## Abbreviations

MRI: magnetic resonance imaging
RIPC: remote ischemic preconditioning
RIPCAGE: Impact of Remote Ischemic Preconditioning preceding Coronary Artery Bypass Grafting on Inducing Neuroprotection Trial
rs-fMRI: resting-state functional magnetic resonance imaging.
